# Supplementary material for: Use of Biolayer Interferometry to Identify Dominant Binding Epitopes of Influenza Hemagglutinin Protein of A(H1N1)pdm09 in the Antibody Response to 2010–2011 Influenza Seasonal Vaccine
Source: Vaccines (Basel). 2023 Jul 31;11(8):1307. doi: 10.3390/vaccines11081307 (PMC10458479; doi:10.3390/vaccines11081307)
Supplement: Supplementary file 1 [file vaccines-11-01307-s001.zip › vaccines-2505571-supplementary.pdf]

Supplementary Materials

Table S1. Age-dependent dominant binding of K130 or K163 in Ab response to pH1N1 vaccine determined by the f-AbBA-2 and the HI assay.

	K130		K163	
**Birth year**	**f-AbBA-2**	**HI**	**f-AbBA-2**	**HI**
1966-1979	1/10 [a]	1/10	6/10	6/10
1981-1991	6/9	4/9	2/9	2/9

[a] Number of post-vaccination serum samples with dominant response to the indicated residue over total number of donors in the age group.

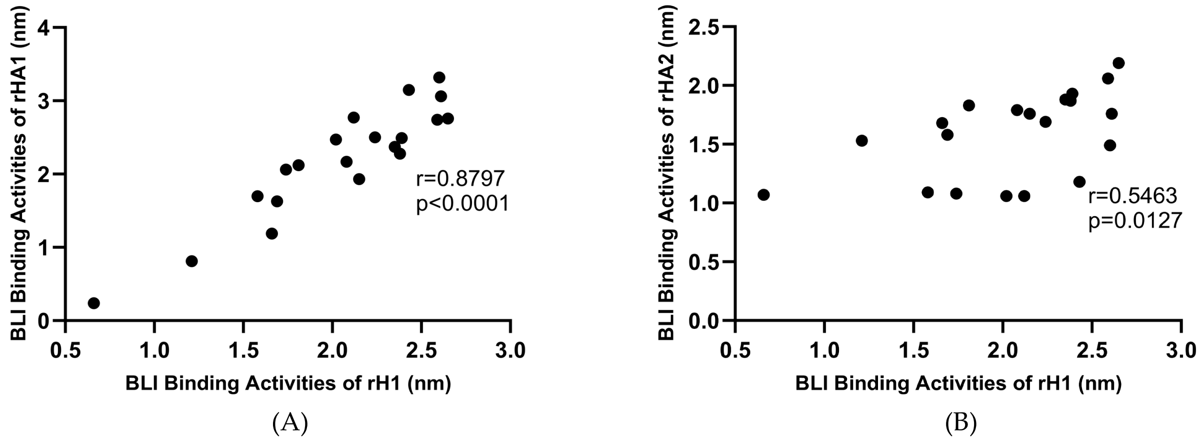

Figure S1. Correlations of the BLI bindings of rH1 to that of rHA1 or rHA2 for the post-vaccination sera. Correlations of the BLI bindings of rH1 to that of rHA1 (**A**) or rHA2 (**B**) for the post-vaccination sera were analyzed. Statistical significance was determined using a two-tailed Spearman correlation coefficient (r) test with r plus *p* values shown.
